# Intense Passionate Love Attenuates Cigarette Cue-Reactivity in Nicotine-Deprived Smokers: An fMRI Study

**DOI:** 10.1371/journal.pone.0042235

**Published:** 2012-07-31

**Authors:** Xiaomeng Xu, Jin Wang, Arthur Aron, Wei Lei, J. Lee Westmaas, Xuchu Weng

**Affiliations:** 1 Department of Psychology, Stony Brook University, Stony Brook, New York, United States of America; 2 Laboratory for Higher Brain Function, Institute of Psychology, Chinese Academy of Sciences, Beijing, China; 3 Graduate University, Chinese Academy of Sciences, Beijing, China; 4 Department of Psychiatry and Human Behavior, Warren Alpert Medical School of Brown University, The Miriam Hospital, Weight Control and Diabetes Research Center, Providence, Rhode Island, United States of America; 5 Mental Health Center of West China Hospital, Sichuan University, Chengdu, China; 6 Behavioral Research Center, American Cancer Society, Atlanta, Georgia, United States of America; 7 Center for Cognition and Brain Disorders, Hangzhou Normal University, Hangzhou, China; Centre for Addiction and Mental Health, Canada

## Abstract

Self-expanding experiences like falling in love or engaging in novel, exciting and interesting activities activate the same brain reward mechanism (mesolimbic dopamine pathway) that reinforces drug use and abuse, including tobacco smoking. This suggests the possibility that reward from smoking is substitutable by self-expansion (through competition with the same neural system), potentially aiding cessation efforts. Using a model of self-expansion in the context of romantic love, the present fMRI experiment examined whether, among nicotine-deprived smokers, relationship self-expansion is associated with deactivation of cigarette cue-reactivity regions. Results indicated that among participants who were experiencing moderate levels of craving, cigarette cue-reactivity regions (e.g., cuneus and posterior cingulate cortex) showed significantly less activation during self-expansion conditions compared with control conditions. These results provide evidence that rewards from one domain (self-expansion) can act as a substitute for reward from another domain (nicotine) to attenuate cigarette cue reactivity.

## Introduction

Cigarette smoking is the number one preventable cause of death in the U.S. [1]. Although medications and behavioral treatments can increase a smoker’s chances of quitting [2], cessation rates have nevertheless stalled [3], standing at around 37% at 6 months post-quit [2]. It is therefore important to investigate new approaches that contribute to understanding tobacco dependence and cessation, and that have the potential to inform strategies to enhance cessation rates.

One approach is that of reward replacement or substitution. Covariation of addiction (or cross-addiction) occurs when common addictive dynamics (e.g., hedonics) substitute for one another [4]–[6]. For example, the physiological effects of alcohol and marijuana are similar, and in some populations (e.g., high school students) efforts to prevent alcohol use have been associated with increases in marijuana use [7], [8]. In addition, rats maintained on a rewarding high-fat diet (vs. low-fat diet) demonstrate decreased cocaine self-administration [9]. Moreover, chronic food restriction has been shown to increase behavioral sensitivity to drugs of abuse [10]. This apparent substitutability may explain why obese people (for whom food reward may be particularly salient) are 25% less likely to develop substance abuse problems [11].

Social reward can also be a strong substitute. Recent studies have found that social interactions can be used as substitutes for food in young adults [12] and that social bonding decreases amphetamine reward (mediated through dopamine) among a monogamous mammalian species, the prairie vole [13]. Social reward in the context of romantic love may also be useful as a substitute. One study of male alcoholics found that among those who ever experienced a period of abstinence, 32% happened to be in the process of beginning a new romantic relationship [14].

Romantic love may act as a strong non-drug reward substitute because it is a very rapid and intense form of “self-expansion.” The self-expansion model proposed by Aron and colleagues [15] posits that people seek to expand the self to increase their physical, informational, and social resources by engaging in experiences that are novel, exciting and interesting. The process of attaining these resources at a rapid rate (e.g., through forming new relationships, taking part in a new hobby, going on a trip, etc.) generates high levels of aroused positive affect and feelings of reward [16]–[17]. Although self-expansion in the context of romantic relationships is typically more rapid and intense during the early stage of the relationship, even among established couples (e.g., those who are married), participating jointly in self-expansion via *exciting* activities (as opposed to pleasant but not particularly exciting activities) is rewarding and significantly increases relationship satisfaction [18].

Self-expansion may also be beneficial during the early stage of smoking cessation because it not only provides reward but has also been shown to mitigate physical pain (through a system different from that of distraction) [19], which could reduce the discomfort associated with withdrawal. Self-expansion also operates on a broader level by changing a person’s sense of identity. In the context of close relationships, as people fall in love they begin to include the other in their sense of self [20]. In non-relationship contexts, as people become immersed in a sport, hobby, etc., which by definition are rewarding, their sense of identity changes to include those aspects. This process by which self-expansion changes one’s sense of identity may also be useful in dealing with addiction, as successful quitters often change their sense of identity from that of a drug user to a healthier self-image [21].

Evidence for the possible role of self-expansion as an aid in smoking abstinence and cessation was found in a study in which smokers who had successfully quit reported experiencing significantly more recent self-expanding events (both social forms of self-expansion as well as self-expansion at the individual level) in their lives prior to their quit attempt compared to smokers who tried to quit but ultimately failed [22]. Even among smokers who attempted to quit but failed, self-expansion experiences were beneficial as there was a significant positive correlation between number of self-expanding events leading up to the quit attempt and how long smokers were able to abstain.

One potential mechanism through which the reward from substances such as nicotine can be replaced by self-expanding events involves the neurotransmitter dopamine, which is linked to reward and motivation [23]–[25]. The brain’s mesolimbic dopaminergic pathway includes the ventral tegmental area (VTA), nucleus accumbens (NAcc), insula, amygdala, medial pre-frontal cortex (mPFC), and dorsal striatum, and plays a key role in addiction [24]–[29] including nicotine addiction [30]–[33]. Although there are many other factors involved in addiction, past research has shown that the hedonic effects of drugs are proportionally related to the amount of dopamine released in the striatum [34]. Some research has shown that when dopamine response is blocked, a corresponding decrease in substance use is observed [35]. As smokers refrain from smoking, craving increases but is attenuated when nicotine is administered [36]–[38].

We posit that self-expansion also activates the mesolimbic dopaminergic system, and in prior research we have demonstrated this in the context of romantic love (particularly during the early-stage). That is, when viewing an image or the name of a romantic partner in an MRI scanner, compared to the image or name of a familiar acquaintance, activation is significantly elevated in regions that include the VTA, caudate, and putamen [39]–[45]. Since nicotine and self-expansion activate the same mesolimbic dopaminergic system, self-expansion may be an appropriate substitute. We were interested in this substitution idea via the mechanism of brain responses during cue exposure.

Past fMRI studies using cigarette cue-reactivity paradigms have found activations in several regions of the brain that include the anterior cingulate cortex (ACC), orbitofrontal cortex (OFC), occipital cortex, parietal cortex, superior frontal cortex (SFC), ventral striatum, thalamus, amygdala, medial frontal gyrus (MFG), posterior cingulate cortex (PCC), cuneus, precuneus, fusiform gyrus, cerebellum and insula [46]–[59]. The current research aimed to investigate whether reward from self-expansion could reduce cigarette cue-reactivity. Specifically, nicotine-deprived smokers in the early stages of love were exposed to smoking or neutral cues while simultaneously viewing images of their romantic partner or a familiar acquaintance. We predicted that self-expansion would lead to less cigarette cue-reactivity activations in the brain. We were particularly interested in six of the more common regions from the cigarette craving-cue literature: ACC, PCC, precuneus, SFC, MFG, and insula.

## Methods

This research was approved by the IRB committees of Stony Brook University and the Chinese Academy of Sciences and all participants provided written informed consent.

### Participants

Participants were 18 Chinese smokers who smoked at least eight cigarettes per day, had been smoking at least 6 months, and who reported being in a non-long-distance romantic relationship with a non-smoker for whom they felt intense passionate love. As the rate of daily smoking is high for men in China (48.9%) and extremely low for women (3.25%), we recruited only men for this study [60]. Participants reported smoking on average 15.78 cigarettes per day (*SD* = 7.83), and that they had been smoking between 6 months and 10 years (*M* = 4.42 years; *SD* = 2.70). Participants were with their partner for an average of 14.22 months (*SD* = 10.97).

Participants were recruited from Beijing campuses by flyers and emails to listservs. Students were targeted for recruitment since they report high rates of new relationships and because this population has been used in previous fMRI studies on romantic love, including one in China [44]. Participants ranged in age from 21–33 (*M* = 25.11, *SD* = 3.03). Participants were excluded if they reported current attempts to quit smoking or using nicotine replacement products. Participants all met the safety protocol for the MRI, were not taking psychoactive medications, and did not have histories of claustrophobia, head trauma, or severe alcohol/drug use (excepting nicotine). All but one preferred their right hand.

### General Procedure

We screened participants over the phone and then invited them to the lab where they completed informed consent and we assessed smoking status with a breath CO monitor [*M* = 14.22 parts per million (ppm), *SD* = 8.77 ppm]. Participants also completed a 14-item version of the Passionate Love Scale [61], where they answered questions on a 1(not true at all) to 9 (definitely true) scale about their partner (e.g., “I have an endless appetite for affection from my partner” and “I would rather be with my partner than anyone else.”) Participants scored an average of 7.75 (SD = .82) on each item in the scale, indicative of intense passionate love.

Participants provided digital photographs of their non-smoker romantic partner and a familiar non-smoker acquaintance (same sex as their partner). Participants were asked to select acquaintances whom they knew for at least as long as their partner and for whom they did not have any romantic feelings or history. Photographs were cropped to show only the head and to ensure similar size. We also asked participants to bring us photographs of a non-smoker close same-sex friend, as we were interested in exploratory analyses with this condition. However, many participants reported after scanning that their close friend was in fact a smoker. Since we wanted to ensure that all face stimuli would not act as a smoking cue, we excluded this condition from our analyses and proceeded with only the partner and familiar acquaintance data.

A separate sample of 7 Chinese volunteers rated all photographs on picture quality on a 1 (extremely bad) to 7 (extremely good) scale. There were no significant differences between partner and acquaintance photographs. Four male volunteers (a subset of the 7) further rated the photographs of the female partners and acquaintances on physical attractiveness on a 1 (absolutely unattractive) to 7 (absolutely attractive) scale. There were no significant differences between partner and acquaintance photographs.

We asked participants to refrain from smoking and nicotine use overnight for at least 8 hours prior to scanning (which took place within 2 weeks of their initial visit). All participants reported abstaining from smoking and nicotine use (including nicotine replacement products) overnight (for at least 8 hours). The majority (all but 2) reported abstaining for at least 12 hours. Immediately prior to scanning, participants had a mean CO measure of 5.83 ppm (*SD* = 2.75), which was a statistically significant drop in ppm from baseline (*M* = 14.22; *SD* = 8.77) *t*(17)  = 9.00, *p*<.001. The average difference in ppm was 8.39 (*SD* = 8.13). Participants also completed a brief version of the Questionnaire of Smoking Urges (QSU-brief) [62]. Participants rated their agreement with 10 statements (e.g., “I have a desire for a cigarette right now”) each on a 0 to 100 rating scale.

All participants were scanned between 2 pm and 6 pm. Prior to scanning, participants were asked to recall memories of their romantic partner and acquaintance. They were told to think of those memories when they saw the corresponding photographs in the scanner, consistent with past research using this paradigm [39], [40], [44]. Also following procedures from previous studies, we instructed participants on a count-back task (mentally counting backwards in increments of sevens). Following the scanning session (which took about 1 hour), participants verbally confirmed that they followed all instructions and completed a post-scan QSU-brief. Participants were then debriefed and given payment of 150 RMB (roughly $25 USD).

### Scanning Stimuli and Procedure

**Figure 1:**
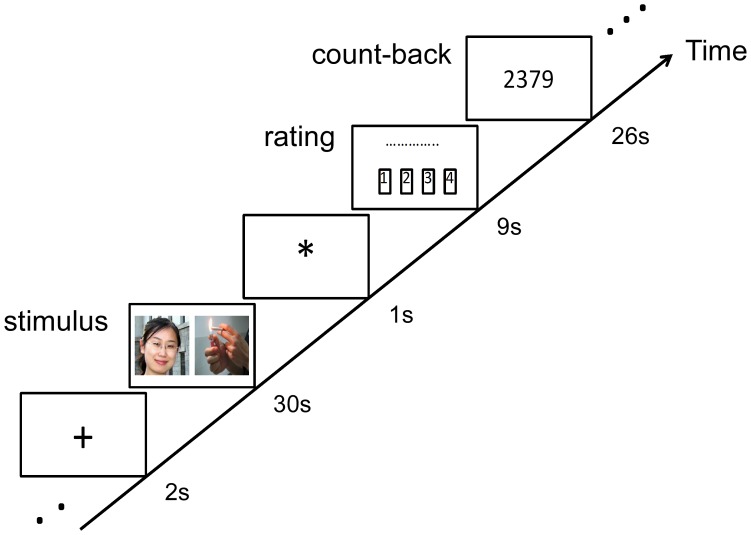
Sample block of experimental task and fMRI design. We used a 2 (partner vs. acquaintance) x2 (cigarette cue vs pen) factor design. Each of the four distinct blocks was repeated three times.

Participants’ data were obtained using a 3T Trio MRI scanner at the Beijing MRI Center for Brain Research. During scanning participants viewed images of people and objects in a 2 (partner vs. acquaintance) ×2 [cigarette cue (i.e., cigarette) vs. pen] block design (see Figure 1). Stimuli images were always viewed in pairs of one person and one object side-by-side (left-right order randomized). Person stimuli were always the same photographs of the romantic partner and acquaintance. Object stimuli were always three images: (a) a hand holding a pen (neutral cue), (b) a hand holding a cigarette (cigarette cue) and (c) a person’s hands as they lighted a cigarette (cigarette cue). We had two different cigarette cues so we could investigate if different cues elicited different levels of cue-reactivity and were affected by self-expansion differently. However we did not find any differences in terms of response to the two cigarette cues and therefore we averaged them together during analyses. Our 2×2 design yielded four pairs of distinct stimuli (partner+cig cue, partner+pen, acquaintance+cig cue, acquaintance+pen), and each pair was repeated three times. Stimuli were presented for 30 s after a 2 s-presentation of a fixation point. Participants were instructed to rate their craving levels for a cigarette for 9 s after the stimulus (the 4 buttons on the response box corresponded with “not at all,” “a little,” “somewhat,” and “extremely”). Due to technical issues during scanning, portions of the rating responses were not recorded for three of our participants. We analyzed the ratings based on the remaining data. Immediately following the ratings, participants completed a count-back task. A random four-digit number appeared on the screen for 26 s and participants mentally counted backwards from that number in increments of 7 s. This count-back task has been used in several other love fMRI studies [39], [44] to help participants disengage from thoughts about the stimuli and to prevent spillover effects across blocks.

All functional scans used a T2* weighted echo planar imaging (EPI) sequence. The imaging parameters were: echo time (TE)  = 30 ms, repetition time (TR)  = 2000 ms, flip angle  = 90°, field of view (FOV)  = 240 mm, and a matrix of 64×64. The whole brain was imaged in an axial configuration where 30 slices were collected and each slice was 3 mm thick (0 mm gap). The resolution was 3.75×3.75×3 mm. After all functional tasks, high resolution anatomical images were collected by using a T1- weighted, three-dimensional gradient-echo sequence (3D MPRAGE) with 144 slices, slice thickness of 1.33 mm, TR of 2530 ms, TE of 3.37 ms, FA of 7°, and a matrix of 256×256, which resulted in a spatial resolution of 1×1.33×1 mm.

### Analyses

We conducted fMRI analyses on data from 17 participants (1 participant’s data were dropped due to a technical problem; no participants’ data needed to be dropped due to excessive motion, which we defined as >2.0 mm translation in any of the three directions or > than 2.0° maximum rotation around any of the axes during the scan). We preprocessed and analyzed the data using AFNI (http://afni.nimh.nih.gov/afni). For each participant, the functional scans were corrected for the slice acquisition timing schedule and head movement, spatially smoothed with a Gaussian kernel of 5 mm FWHM and normalized so that each voxel time series would have a mean of 100. Single-subject whole brain voxel size General Linear Model (GLM) analyses were performed to estimate the individual statistical *t*-maps. There were ten regressors in the GLM in all, with four regressors representing four experimental conditions (partner+cig; partner+pen; acquaintance+cig; acquaintance+pen) and the six head-motion parameters. The regression analysis incorporated correction for the temporal autocorrelation of voxelwise noise (AFNI program 3dREMLfit).

Group analyses were preformed after converting functional images into Talairach space (re-sampled to a voxel size of 3×3×3 mm^3^). We compared activations across participants using AFNI program 3dANOVA3 (two-way mixed factor) with condition as the fixed factor and participants as the random factor for each group. A group statistical map was created with four contrasts: partner+cigarette vs. partner+pen (which assesses cigarette cue reactivity in the presence of self-expansion reward); acquaintance+cigarette vs. acquaintance+pen (which assesses cigarette cue reactivity in the absence of self-expansion reward); partner+cigarette vs. acquaintance+cigarette (which assesses the self-expansion effect in the presence of cigarette cues) and partner+pen vs. acquaintance+pen (which assesses the self-expansion effect in the absence of cigarette cues). To correct for multiple comparisons, statistically defined clusters of activation were identified using whole-brain Monte Carlo simulation (AFNI program Alpha Sim) to achieve a corrected cluster threshold of *p*<0.05.

**Table 1:** Regional activations and deactivations for cigarette-cue contrasts.

	High craving smokers					Moderate craving smokers				
	Peak *x*	Peak *y*	Peak *z*	Peak *t*-value	Voxels	Peak *x*	Peak *y*	Peak *z*	Peak *t*-value	Voxels
**Partner-cig vs. Partner-pen**										
*Activations*										
left posterior cingulate						−6	−57	16	9.14	118
left middle temporal gyrus						−40	−64	13	5.29	69
*Deactivations*										
right precuneus	14	−70	41	−7.04	461					
left inferior parietal gyrus	−46	−43	41	−7.70	342					
left inferior frontal gyrus	−46	2	31	−10.56	171					
left insula	−43	−37	20	−6.89	90					
**Acquaintance-cig vs. Acquaintance-pen**										
*Activations*										
bilateral posterior cingulate						−1	−61	17	11.19	556
left supramarginal gyrus						−49	−55	32	8.36	140
right medial frontal gyrus						8	44	14	6.64	81
left superior frontal gyrus						−16	47	26	6.28	71
left anterior cingulate						−10	44	9	4.63	67
*Deactivations*										
left superior parietal lobule	−31	−73	50	−5.09	154					
right superior parietal lobule	41	−58	50	−4.45	112					

Whole brain analyses results for cigarette-cue contrasts. Coordinates are Talairach. We accepted p<0.05 (FWE-corrected) for the single peak voxel in a cluster with a minimum of 65 voxels (high craving group) and 67 voxels (moderate craving group). We also highlight several smaller clusters of interest that did not meet the minimum voxel size set by our Monte Carlo simulations. These smaller clusters were identified using a threshold of p<0.01 uncorrected, and we use an * in this table to indicate these smaller clusters of interest.

**Table 2:** Regional activations and deactivations for partner-cue contrasts.

	High craving smokers					Moderate craving smokers				
	Peak x	Peak y	Peak z	Peak t-value	Voxels	Peak x	Peak y	Peak z	Peak t-value	Voxels
**Partner-pen vs. Acquaintance-pen**										
*Activations*										
left putamen						−25	2	17	8.23	23*
right culmen						23	−43	−18	4.51	20*
right inferior parietal lobule						41	−34	41	9.50	19*
right middle frontal gyrus						38	17	23	6.17	15*
left caudate head						−13	11	2	7.25	12*
left middle frontal gyrus						−25	−10	41	7.27	12*
right caudate head						11	14	6	8.28	11*
left cingulate gyrus	−13	−28	32	5.74	10*					
*Deactivations*										
right anterior cingulate	14	41	−3	−5.44	10*					
**Partner-cig vs. Acquaintance-cig**										
*Activations*										
left middle frontal gyrus						−40	31	26	6.98	19*
white matter	−25	−40	20	5.83	84					
*Deactivations*										
left cuneus						−4	−88	14	−6.39	48*
right superior temporal gyrus	56	−28	14	−5.28	75					
left amygdala	−14	2	−12	−7.39	65					

Whole brain analyses results for partner-cue contrasts. Coordinates are Talairach. We accepted p<0.05 (FWE-corrected) for the single peak voxel in a cluster with a minimum of 65 voxels (high craving group) and 67 voxels (moderate craving group). We also highlight several smaller clusters of interest that did not meet the minimum voxel size set by our Monte Carlo simulations. These smaller clusters were identified using a threshold of p<0.01 uncorrected, and we use an * in this table to indicate these smaller clusters of interest.

For region of interest (ROI) analyses we were interested primarily in two regions associated with self-expansion reward, namely the caudate and VTA, as well as several regions associated with cigarette cue-reactivity, namely the PCC, ACC, insula, precuneus, MFG, and SFC. The ROIs were defined functionally as spheres with a 6-mm radius (3 mm for the VTA) on the basis of activation clusters from the group analyses. The peak activation coordinates from the cluster of the contrast analysis were selected as the center of each ROI. We built ROIs around coordinates for caudate and VTA from a prior love fMRI study [44] and cue-reactivity regions from the whole brain analyses in our two groups. We averaged the signal for voxels in each ROI using the AFNI program 3dmaskave. We converted regression coefficients to percent signal change for each ROI for each condition, and used SPSS 17.0 to run repeated measures ANOVAs to compare percent signal change in our contrasts.

**Figure 2:**
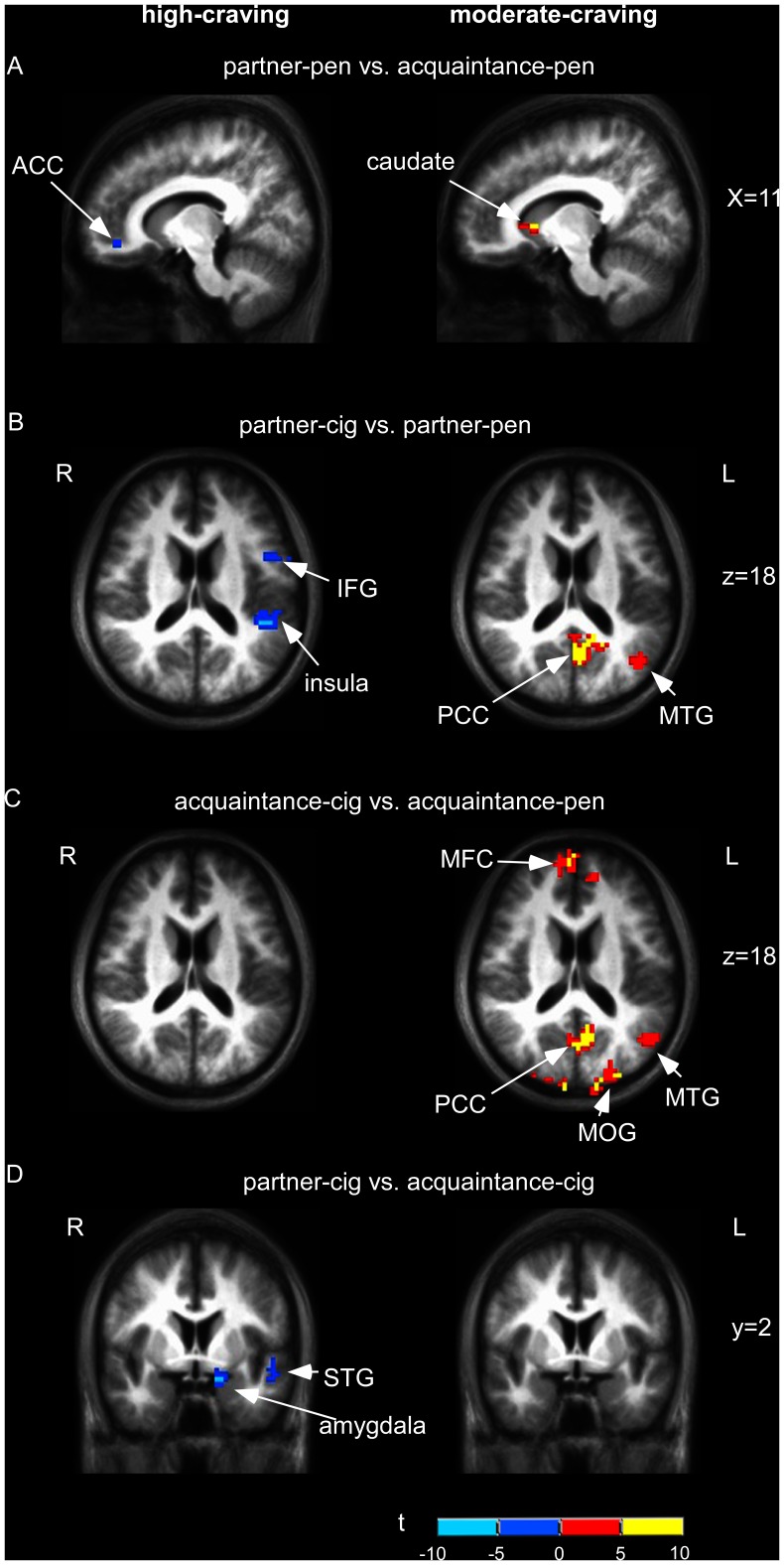
Whole brain comparisons between conditions. A: partner+pen vs. acquaintance+pen (p<0.01 uncorrected); B: partner+cig vs. partner+pen (IFG) (p<0.05, corrected); C: cue-induced craving: acquaintance+cig vs. acquaintance+pen (p<0.05, corrected); D: partner+cig vs. acquaintance+cig (p<0.05, corrected).

## Results

Self-reported ratings of cravings did not differ between pen versus cig-cue presentations. As we asked participants to rate their craving every 30 seconds (no prior overnight abstinence study has asked for reporting cravings with this much frequency), and on a scale that only offered 4 options, this rating system may not have been sensitive enough to pick up on variability in subjective craving. Participants differed markedly, however, on self-reported craving levels (QSU-brief scores) immediately prior to entering the scanner, with some participants reporting elevated levels of cravings. To thus address the role of potential ceiling effects on cue-reactivity, we divided participants into two groups. We labeled as our “moderate craving” group those with QSU-brief scores <400 (800 was the highest score in our sample; n = 7; *M* = 308±95). We labeled as our “high craving” group those with scores >400 (n = 10; *M* = 631±133). Asians are less likely to use the higher anchors on the QSU-Brief scale which leads to overall lower raw QSU-Brief scores compared to Western samples undergoing nicotine abstinence [63], [64]. There were no significant differences by group in terms of length of romantic relationship, number of cigarettes smoked per day, or number of years as a smoker. Those in the high-craving group were significantly older (*M* = 26.45 years, *SD* = 2.95 vs. *M* = 23 years, *SD* = 1.7, *p* = .013). There was a trend such that the high-craving group showed a greater reduction in carbon monoxide levels from baseline to pre-scan (*M* = 11.09, *SD* = 8.98 vs. *M* = 4.14, *SD* = 4.30; *p* = .076). The high-craving group also seemed to have reached a craving ceiling, such that their pre-scan QSU-brief scores did not differ significantly from their post-scan scores (*p* = .761). However, the moderate-craving group had significantly higher post- than pre-scan QSU-brief scores, *t*(6)  = −2.73, *p* = .034.

**Figure 3:**
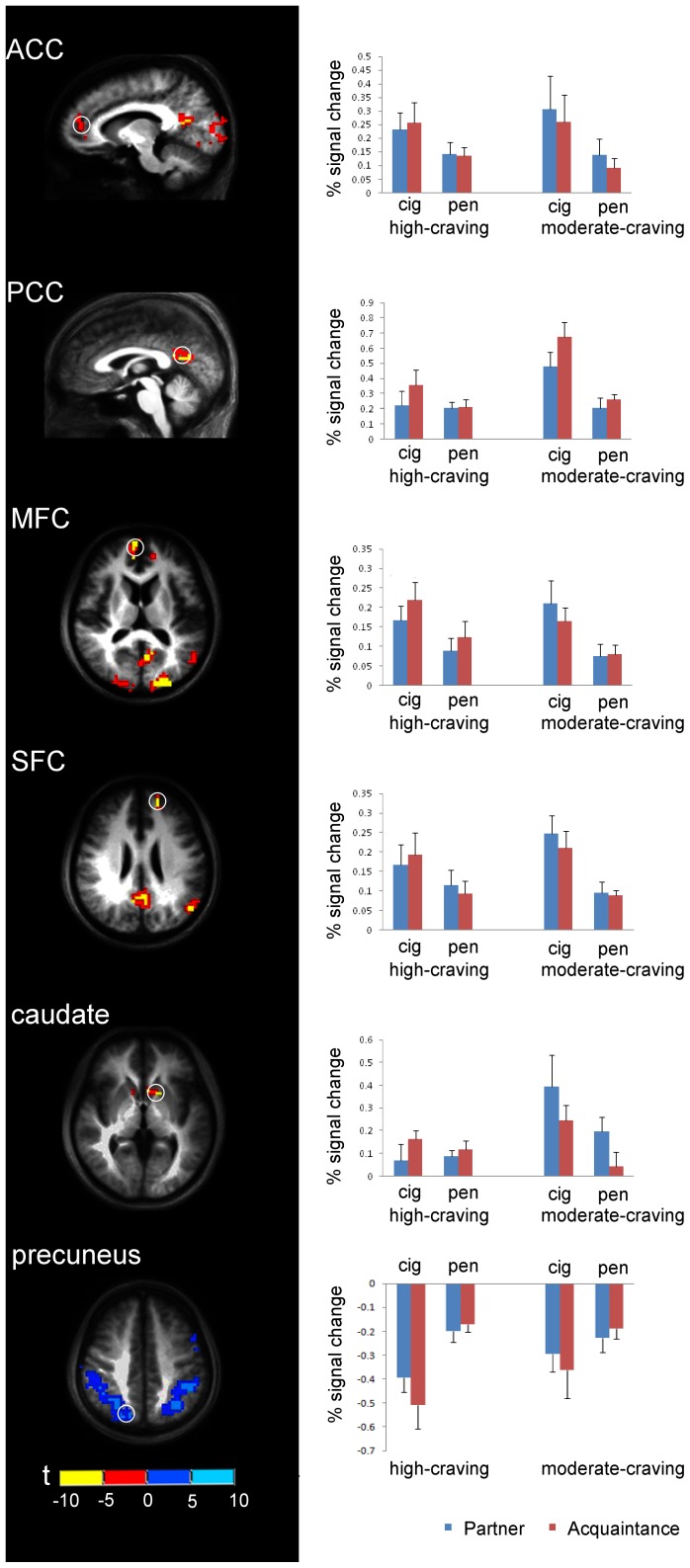
Region of Interest (ROI) analysis of the percent signal change of six ROIs for the four experimental conditions (partner+cig, partner+pen, acquaintance+cig, and acquaintance+pen). The radius for each ROI sphere is 6 mm, VTA was 3 mm. The left line shows the location of each ROI (white circle), the right line shows the corresponding percent signal change for four experimental conditions. The center coordinates (Talarich coordinates)of each ROI sphere are: anterior cingulate cortex(ACC)(-10, 44, 9); posterior cingulate cortex(PCC)(-1,-61,17); middle frontal cortex(MFC)(8,44,14); superior frontal cortex(SFC)(-16,47,26); caudate(-13,11,2) and precuneus(14–70 41). The ROI regions ACC, PCC, MFC and SFC are defined from an activation map contrasting acquaintance+cig vs. acquaintance+pen in moderate-craving group; caudate is defined from an activation map of partner+pen vs. acquaintance+pen in moderate-craving group and precuneus is defined from an activation map of partner+cig vs. partner+pen in high-craving group.

### Whole Brain Analyses

To correct for multiple comparisons, Monte Carlo simulation was used to achieve a corrected cluster threshold of *p*<0.05, which yielded corrected clusters reaching contiguous volumes of at least 65 voxels for the high-craving group and 67 voxels for the moderate-craving group, with a voxel-wise threshold of *p*<0.01. These cutoffs yielded large clusters. However, we were also interested in smaller regions associated with cue-reactivity that may still be meaningful (despite not meeting statistical significance via the Monte Carlo simulations), and set a threshold of *p*<0.01 uncorrected for exploring these smaller clusters of voxels (see Tables 1 & 2).

#### Manipulation checks

To verify cigarette cue-reactivity, whole brain analyses compared cigarette cues with neutral cues in acquaintance conditions. In the moderate-craving group, cigarette cue conditions compared to the neutral cue conditions, elicited activations in cue-reactivity regions, specifically the bilateral posterior cingulate, bilateral middle occipital gyrus, right media frontal gyrus, left superior frontal gyrus, left anterior cingulate gyrus, left supramarginal gyrus and left middle temporal gyrus (*p*<0.05 corrected). In the high-craving group, cigarette cues (vs. neutral cues) were associated with deactivation in the bilateral superior parietal gyrus, left precuneus and right supramaginal gyrus. To examine if there was a reward effect of self-expansion independent of smoking-related cues, we compared activations of partner versus acquaintance presentations accompanied by neutral cues. In the moderate-craving group, there were activations in the left putamen, bilateral caudate, bilateral middle frontal gyrus, right inferior parietal lobule and right culmen (*p*<0.01 uncorrected); in the high-craving group, in the left cingulate gyrus and deactivation in the right anterior cingulate (*p*<0.01 uncorrected).

#### Cue-reactivity and self-expansion results

For the partner+cig cue vs. acquaintance+cig cue contrast, in the moderate-craving group, we found deactivation in the left cuneus (*p*<0.01 uncorrected; see Table 2), and for the high-craving group deactivation in the left amygdala (*p*<0.01 uncorrected; see Table 2, Figure 2). As a further test, we compared results between the partner+cig vs. partner+pen contrast and the acquaintance+cig vs. acquaintance+pen contrast. Moderate-craving smokers, when exposed to images of their partner and a cigarette cue (vs. partner and a neutral cue), showed activation in the left posterior cingulate gyrus and left middle temporal gyrus (*p*<0.05 corrected), regions also activated in the acquaintance+cig vs. acquaintance+pen contrast. However, as predicted, there was more activation in these regions in the acquaintance+cig vs. acquaintance+pen contrast compared to the partner+cig vs. partner+pen contrast (see Table 1; Figure 2). For high-craving smokers, partner+cig vs. partner+pen yielded deactivations in bilateral precuneus, left inferior frontal gyrus and left insula (*p*<0.05 corrected), while acquaintance+cig vs. acquaintance+pen yielded deactivations in bilateral parietal lobule, an area associated with somatosensory function (*p*<0.05 corrected. See Table 1; Figure 2).

### ROI Analyses

#### Manipulation check

We found a significant cigarette cue-reactivity effect for our acquaintance conditions (acquaintance+cig vs. acquaintance+pen) in the ACC (center coordinates: −10, 44, 9; *p* = .001), PCC (center coordinates: −1,−61, 17; *p*<.001), MFC (center coordinates: 8, 44, 14; *p* = .002), and SFC (center coordinates: −16, 47, 26; *p*<.001). Similar to our whole-brain analyses, we found these activations more robustly for moderate-craving smokers compared to high-craving smokers (i.e., interaction effects). Specifically, although for both moderate- and high-craving smokers there was significant cigarette cue-reactivity activation in the MFC and SFC during acquaintance conditions, the effect was stronger in the moderate-craving group for activation in the PCC (*p* = .010) and marginally stronger in the ACC (*p* = .062) (see Figure 3). Across both moderate- and high-craving groups we obtained cigarette vs. neutral cue deactivation in the precuneus (center coordinates 14, -70, 41; *p* = .047), but an interaction effect approached significance (*p* = .065) indicating less deactivation in moderate-craving smokers compared to high-craving smokers (see Figure 3).

For the self-expansion reward manipulation check contrast we found significantly more activation in the caudate (center coordinates −13, 11, 2) for partner conditions compared to acquaintance conditions, *p* = .021, with the partner x craving-group interaction approaching significance, *p* = .079. (see Figure 3).

#### Cue-reactivity and self-expansion results

There was a significant cue x self-expansion interaction in the PCC (*p* = .046), such that when moderate-craving smokers viewed cigarette cues alongside images of their partner, there was less activation in the PCC compared with when they viewed cigarette cues alongside images of an acquaintance (see Figure 3).

## Discussion

The present experiment used fMRI to examine whether self-expansion through the reward of romantic attraction could decrease brain responses to cigarette cues among nicotine-deprived smokers. A manipulation check confirmed that our smoking cues (compared to neutral cues) yielded significant activation in cue-reactivity regions, notably the anterior and posterior cingulate cortex and prefrontal cortex, during exposure to images of an acquaintance. Moreover, during exposure to images of a romantic partner, results indicated that smoking cues (compared to neutral cues) were associated with deactivation in cue-reactivity regions. This suggests that the reward associated with self-expansion experiences can help attenuate cue reactivity. However, these effects were robust primarily among smokers for whom cravings were not so high that they overwhelmed the effect of self-expansion. For smokers who were experiencing higher levels of cigarette craving prior to scanning, cigarette cues did not elicit more brain response in cue-reactivity regions than control images. Moreover, partner images for these smokers did not elicit more reward-motivated activations than acquaintance images. This indicated that a craving ceiling had been reached such that the effects of cigarette cues and self-expansion were not evident.

We also obtained an unexpected finding of significantly more deactivation in the precuneus in cigarette-cue conditions compared to control conditions. While the precuneus has been an area associated with cue reactivity, our deactivation results may actually be reflecting a different portion of the precuneus, one that includes the inferior parietal lobule, an area associated with internal representations [65]. This suggests that participants were focusing less internally during cigarette cue conditions (perhaps attending to the cigarette cue more) compared to control conditions.

Even among moderate-craving smokers (who had not reached a craving ceiling), although partner images still yielded some reward activation (in the caudate), we did not find expected VTA activation. The VTA is an area especially sensitive to intense romantic love reward, whereas the caudate is a region associated with social reward more generally including positive romantic relationships past the initial intense stage [41]. No other neuroimaging study of love has focused on smokers or investigated participants who had been asked to abstain from any substance, so it is unclear if the lack of significant VTA activations is a finding unique to smokers. It is also possible that while the reward of self-expansion attenuates cigarette cue reactivity (as we found), overnight abstinence and cigarette cue reactivity may also interfere with self-expansion reward associated with the VTA. Future studies, including studies with non- nicotine-deprived smokers, could further explore this potential bidirectional relationship to better understand the threshold at which one effect trumps the other, and various potential moderating and mediating factors.

There were some limitations to our study and thus the generalizability of our results. First, because we followed the procedures of previous fMRI studies of romantic love [39], [40], [44], we used only one image of the partner and one image of the acquaintance and as a consequence also used the same cigarette and pen cues repeatedly. Although past fMRI love studies have found no habituation effects, this is the first study focusing on smokers, and in particular, smokers who have undergone overnight abstinence. It is possible that habituation effects were present for this sample and influenced the results (although this would presumably function primarily to weaken our results). Future studies could use multiple stimuli to ensure that habituation does not occur. Second, we did not find cue-reactivity activations in our sample for several regions that have been implicated in prior literature, particularly in the limbic system. Some potential reasons for this include the design (e.g., possible habituation effects as just noted) as well as the possibility that because smokers abstained overnight, they may have been in withdrawal such that they were exhibiting ceiling effects on limbic responses prior to the cue protocol.

The current study did not include a distraction control condition, which might be an alternative explanation for our results (however, in the study using a similar paradigm to test effects of self-expansion on pain, [19], there was similar pain reduction for both distraction and viewing the beloved, but they operated through different neural systems). In addition, the current study did not have an aversive control condition to specifically test if aversive arousing stimuli would have equally attenuated cue-reactivity. However, brain regions associated with arousing aversive stimuli, for example the amygdala [66], did not show activations in our experimental conditions, leading us to conclude that this alternative explanation is unlikely.

Finally, our sample consisted of current smokers who were not in the process of quitting. For purposes of directly applying our results in intervention settings, future studies should investigate our model among smokers who are attempting to quit to test cue-reactivity reduction in a more clinically relevant sample, and to investigate if quit readiness moderates the effect of self-expansion. However, previous fMRI studies on cigarette cue-reactivity have found activations in similar regions for both non-treatment seeking and treatment seeking smokers, suggesting that our model may be appropriate even for those attempting to quit [50], [56], [67], [68]. Future studies are necessary to directly investigate this.

The overall results of this research advances our general understanding of reward and cue-reactivity processes in the context of addiction and self-expansion. This research also advances our understanding of self-expansion as a novel approach (one that has never been tested empirically before except by self-report measures) to undermining cigarette cue-reactivity. As an initial study of self-expansion effects, we focused on romantic love because it is one of the most robustly intense and rapid forms of self-expansion and one that is testable in a research situation. However, now that effects have been observed under these strong conditions future research could focus on less intense but widely experienced (and more practical as intervention methods) self-expansion activities/events by including both other social self-expansion experiences (e.g., interactions with friends and family members), as well as self-expansion at the individual level (e.g., engaging in a new sport or hobby or engaging in spiritual experiences). Sport and exercise might be an especially fruitful area as exercise has been shown to help with cigarette craving [69] and to increase dopamine [70]. Mindfulness practices may also be especially interesting to investigate as self expansion can be a mediator for mindfulness [71] and mindfulness has been linked to reductions in smoking [72], [73] and lower levels of nicotine dependence and withdrawal severity [74]. Finally, future studies utilizing self-expansion could build upon these results to help create an intervention for smoking cessation and to investigate if a self-expansion model could be applied to other addictions as well.
